# Compact Nonlinear Yagi-Uda Nanoantennas

**DOI:** 10.1038/srep18872

**Published:** 2016-01-07

**Authors:** Xiaoyan Y. Z. Xiong, Li Jun Jiang, Wei E. I. Sha, Yat Hei Lo, Weng Cho Chew

**Affiliations:** 1 Department of Electrical and Electronic Engineering, The University of Hong Kong, Pokfulam Road, Hong Kong; 2 Department of Electrical and Computer Engineering, The University of Illinois at Urbana-Champaign, Illinois 61801, USA

## Abstract

Nanoantennas have demonstrated unprecedented capabilities for manipulating the intensity and direction of light emission over a broad frequency range. The directional beam steering offered by nanoantennas has important applications in areas including microscopy, spectroscopy, quantum computing, and on-chip optical communication. Although both the physical principles and experimental realizations of directional linear nanoantennas has become increasingly mature, angular control of nonlinear radiation using nanoantennas has not been explored yet. Here we propose a novel concept of nonlinear Yagi-Uda nanoantenna to direct second harmonic radiation from a metallic nanosphere. By carefully tuning the spacing and dimensions of two lossless dielectric elements, which function respectively as a compact director and reflector, the second harmonic radiation is deflected 90 degrees with reference to the incident light (pump) direction. This abnormal light-bending phenomenon is due to the constructive and destructive interference between the second harmonic radiation governed by a special selection rule and the induced electric dipolar and magnetic quadrupolar radiation from the two dielectric antenna elements. Simultaneous spectral and spatial isolation of scattered second harmonic waves from incident fundamental waves pave a new way towards nonlinear signal detection and sensing.

Optical nanoantennas, analogous to microwave and radio frequency antennas, convert freely propagating optical radiation into localized electromagnetic energy, and vice versa^1^,^2^,^3^,^4^. They have broad and rich applications in photodetection, sensing, microscopy, spectroscopy, quantum computing, optical communications, etc 5,6,7,8,9,10,11. Nanoantennas featuring compact dimensions and high directivities are promising building blocks to enhance light-matter interaction at subwavelength scales. Considerable efforts are focusing on the angular control of light emission via plasmonic, dielectric, and composite nanostructures^9^,^10^,^12^,^13^. However, compared to its linear counterparts, tailoring the radiation pattern of nonlinear sources is full of great challenges. First, nonlinear radiation exhibits complex multipolar (dipolar, quadrupolar, etc) interactions caused by both surface and bulk nonlinear susceptibility tensors^14^,^15^,^16^,^17^. Second, modeling nonlinear scattering processes in complex geometries lacks an efficient and rigorous simulation tool. Third, physical principles and designs for beam steering of nonlinear nanoantennas have not been explored yet, although enhancement mechanisms of nonlinear signals have been extensively studied^18^,^19^,^20^,^21^,^22^,^23^,^24^,^25^,^26^,^27^,^28^.

Here, we propose a novel nonlinear Yagi-Uda nanoantenna concept. The Yagi-Uda antenna is one of the most brilliant ideas of directional antennas^29^,^30^,^31^,^32^,^33^,^34^. It has been explored for directionally controlling electromagnetic radiation at both microwave and optical frequencies. In this work, a Yagi-Uda nanoantenna is employed to steer second harmonic generation from a metallic nanosphere that behaves as a point-like feed. Two high-refractive-index dielectric nanoparticles (elements) with very low dissipative losses are engineered to form a compact director and reflector^34^. Through optimization of the spacing and dimensions of the two dielectric elements, the resultant nonlinear nanoantenna obtains constructive interference of second harmonic waves in one direction and destructive interference in the opposite direction. Interestingly, the second harmonic radiation is bent at 90 degrees with respect to the direction of incident fundamental pump. This abnormal radiation behavior, which cannot be achieved using linear Yagi-Uda nanoantennas, is attributed to a special selection rule of second harmonic radiation and an induced phase shift between the reflector and director. Although the Kerr effect^35^,^36^ has been explored to control the far-field radiation of nonlinear antennas, the design of the nonlinear antennas is similar to that of linear counterparts due to the dominating local-bulk dipolar mode in small metallic nanoparticles. Different from the Kerr effect, the second harmonic radiation from the nanoparticle shows a complex surface-induced multipolar interaction without the local-bulk dipolar mode. Moreover, the far-field radiation obeys a number of selection rules strongly relying on the symmetry and surface characteristics of the nanoparticle^37^. Due to spatial and spectral separations between fundamental fields and second harmonic fields, our work opens up a hopeful route to nonlinear detection, sensing, and signal manipulation.

## Results

### Theoretical modeling for second harmonic radiation

The mutual interaction between the fundamental fields and second harmonic fields can be described by the following coupled wave equations^38^


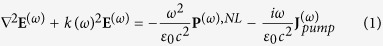



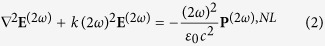


where 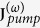 is a pump source producing the incident fundamental field. The nonlinear polarizations 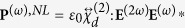 and 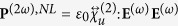 are the source terms of the fundamental fields and second harmonic fields. The second-order nonlinear susceptibilities related to the down-conversion and up-conversion processes are respectively defined as 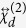 and 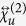. 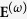 and 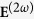 denote the fundamental and second harmonic electric fields, respectively. 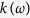 and 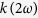 are the corresponding wave numbers. To fully capture the pump depletion effect and cross coupling between the fundamental fields and second harmonic fields, equations (1) and (2) will be solved self-consistently using a rigorous boundary element method^16^,^39^,^40^ with an initial condition of 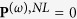. Regarding expanded simulation details, please see Methods Section.

### Second harmonic radiation from a metallic nanosphere

**Figure 1:**
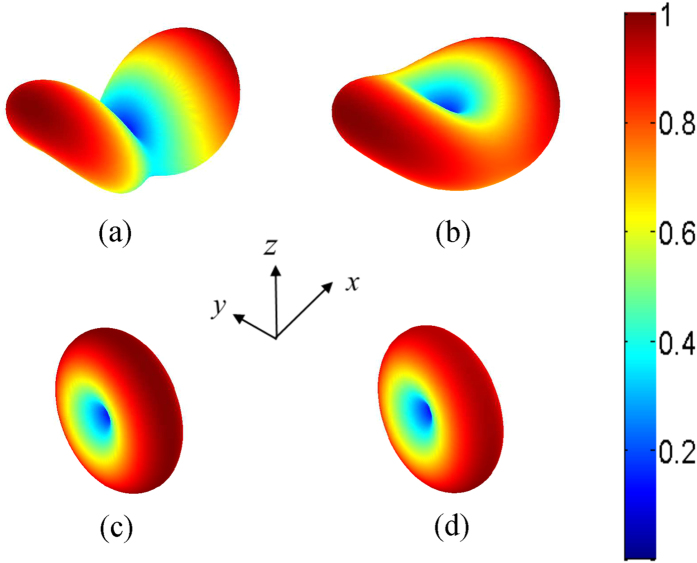
Normalized radiation pattern of a gold nanosphere with a radius of 20 nm. The incident fundamental pump is an *x* polarized plane wave propagating along the *z* direction. (**a,b**) second harmonic radiation patterns; (**c,d**) fundamental radiation patterns. (**a,c**) correspond to the pump wavelength of 520 nm. (**b,d**) correspond to the pump wavelength of 780 nm.

Typically, both bulk and surface nonlinear sources contribute to the second harmonic radiation. However, for metal materials with centrosymmetric crystal structures, the local-bulk source is absent. Only the nonlocal-bulk sources including a negligible term and surface-like bulk term^17^ exist. Owing to symmetry breaking and plasmonically induced field localization at the interface, the surface contribution presumably dominates the second harmonic radiation from a metallic nanostructure. Figure 1(a,b) shows the second-harmonic radiation pattern of a gold nanosphere with a radius of 20 nm. Compared to the fundamental far-field scattering as illustrated in Fig. 1(c,d), the second harmonic radiation obeys a special selection rule^41^ that the radiation is strictly zero along the direction of an incident pump (plane wave). In contrast to the perfect dipolar pattern of the fundamental scattering [See Fig. 1(c,d)], another unique feature of the second harmonic radiation is a complex multipolar interaction. If an incident pump occurs at the plasmonic resonant frequency of the nanosphere (520 nm), both the dipolar and quadrupolar moments contribute to the far-field second harmonic radiation [See Fig. 1(a)]. If the incident pump is off the plasmonic resonance (780 nm), the second harmonic radiation shows a dipolar pattern slightly distorted by a weak quadrupolar contribution [See Fig. 1(b)]. Although the plasmonic resonance will significantly enhance second harmonic generation, detection of the second harmonic signals at 260 nm is tremendously difficult due to the strong ultraviolet absorption of silica. Hence, this study only considers the off-resonance case, where both the fundamental pump (780 nm) and second harmonic radiation (390 nm) frequencies fall into the visible light regime. These conditions offer favorable convenience for both photodetection and post-processing. Furthermore, the dipole-like radiation pattern, shown in Fig. 1(b), enables the unidirectional emission of second harmonic signals, which will be discussed in the following sections.

### Design of dielectric reflector and director elements

**Figure 2:**
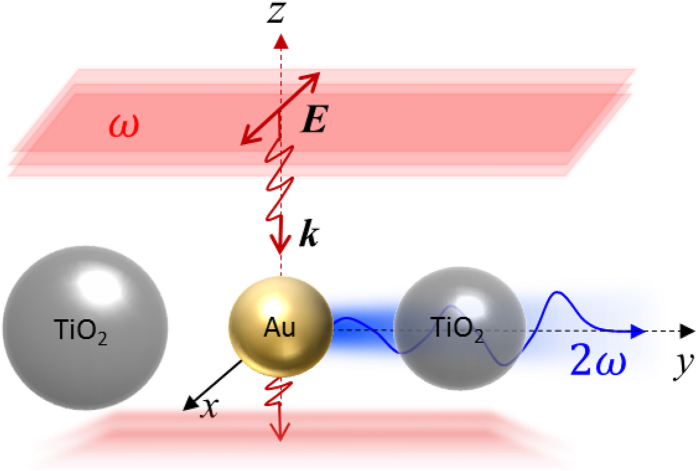
The schematic pattern of a unidirectional, compact and nonlinear Yagi-Uda nanoantenna. The incident pump (with a red wavefront) is an *x* polarized plane wave propagating along the *–z* direction. A metallic (Au) nanosphere radiating second harmonic waves (denoted by blue color) can be regarded as a dipole-like feed. The left and right dielectric (TiO_2_) spheres, working respectively as the reflector and the director, realize the unidirectional radiation of second harmonic waves. The second harmonic radiation is bent at 90 degrees with reference to the incident pump direction. The optimized radii for feed, reflector, and director are 20 nm, 80 nm and 70 nm, respectively. The spacing between the metallic feed and dielectric elements are 50 nm.

**Figure 3:**
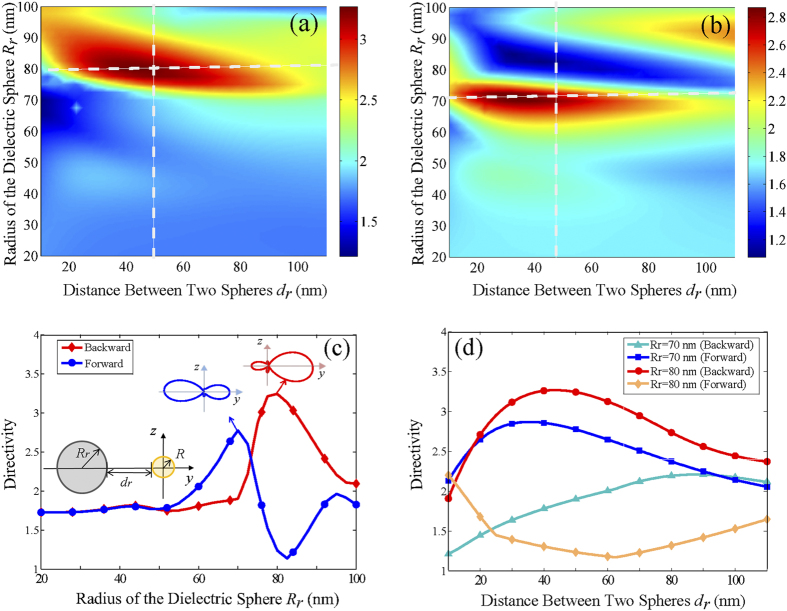
Forward and backward directivities of second harmonic radiations as a function of the radius *R*_*r*_ of the dielectric sphere and the distance *d*_*r*_ between the dielectric and metallic spheres. (**a**) backward directivity; (**b**) forward directivity; (**c**) forward and backward directivities as a function of the radius *R*_*r*_ with a fixed distance *d*_*r*_ of 50 nm; (**d**) forward and backward directivities as a function of the distance *d*_*r*_ with fixed radii.

Figure 2 schematically illustrates the proposed unidirectional, compact, and nonlinear Yagi-Uda nanoantenna. An *x*-polarized plane wave with a wavelength of 780 nm propagates along the *-z* direction to illuminate the nanoantenna. A gold nanosphere with a radius of 20 nm, acts as a point-like feed, producing a second harmonic wave at 390 nm. The second harmonic wave is then redirected and steered by two high-refractive-index lossless dielectric spheres (TiO_2_), which function as the reflector and the director elements of the nanoantenna. The compact configuration occupies only 440 nm in the *y* direction, allowing the three elements to interact in near-, intermediate-, and far-field regimes simultaneously. To achieve a high directivity for the second harmonic radiation, sizes of the reflector and the director and their distances to the feed (metallic sphere) must be carefully engineered and fully optimized. To understand their working mechanism, one can consider a simplified two-sphere system including a dielectric element and a metal feed placed along the *y* axis. Figure 3(a,b) depicts forward (−*y*) and backward (+*y*) directivity maps for the second harmonic radiation, as a function of the dielectric sphere radius and the spacing between the dielectric and metallic spheres. The dielectric sphere can be varied, allowing it to function as a director or reflector element, depending on the magnitudes of forward directivity 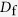 and backward directivity 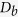 defined as


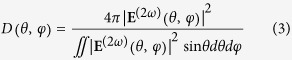



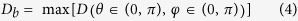



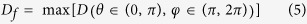


**Figure 4:**
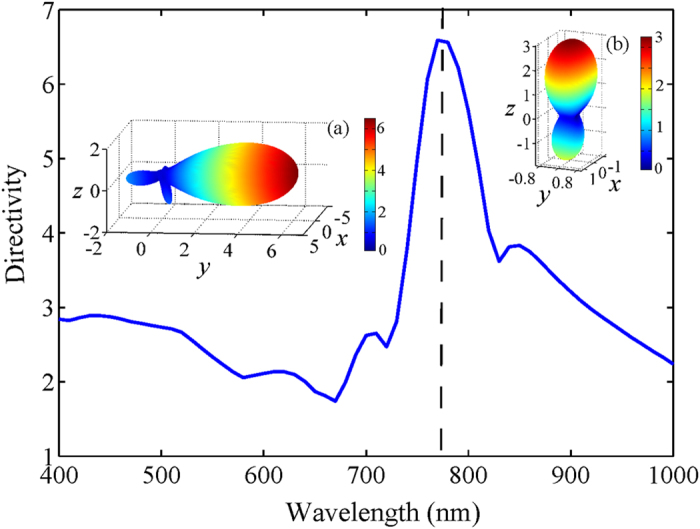
Directivity of the optimized nonlinear Yagi-Uda nanoantenna as a function of the pump wavelength. The insets show the radiation pattern of the nanoantenna at the pump wavelength of 780 nm. (**a**) second harmonic radiation pattern; (**b**) fundamental radiation pattern.

Here, *θ* and *φ* are spherical angles. On one hand, the radius will strongly affect the mutual interference of the multipolar resonances in the dielectric sphere. On the other hand, a specific degree of field retardation, tunable with the spacing between the dielectric and metallic spheres, is essential to the phase control of induced multipoles inside the dielectric sphere. Accordingly, dielectric spheres with a radius of 70 nm (with a high forward directivity) and 80 nm (with a high backward directivity) are selected, respectively, for the director and reflector elements [See Fig. 3(c)]. To realize a compact structure, elements of larger radii were not investigated, though that may induce a stronger directivity. The near- and intermediate-field couplings between the metallic and dielectric spheres play an important role in enhancing the directivity as presented in Fig. 3(d). An optimized spacing around 50 nm is preferred for achieving a desired angular control. Integrating the dielectric reflector and director with the metallic feed in this geometry, the composite nonlinear Yagi-Uda nanoantenna gains a high directivity of 6.5 for the second harmonic radiation as illustrated in Fig. 4. It should be noted, according to the selection rule and the corresponding far-field pattern in Fig. 1 (b), the second harmonic scattering from the feed can be considered approximately as a *z*-polarized Hertzian dipole source generated by the nonlinear upconversion process. Secondly, the omni-directional second harmonic scattering from the dipole source will be reshaped as a unidirectional second harmonic radiation after employing the optimized director and reflector. Finally, the second harmonic radiation is bent at 90 degrees with respect to the incident pump direction as presented in Fig. 2 and 4(a). The abnormal second harmonic radiation, which is fundamentally different from the radiation of linear nanoantennas as indicated in Fig. 4(b), is undoubtedly observed in the nonlinear Yagi-Uda nanoantenna. Thanks to this abnormal radiation feature, spectral and spatial isolations between the fundamental and second harmonic waves are successfully realized simultaneously. In the next section, we will elucidate the complex electromagnetic interaction between the three antenna elements to unveil the physical origin of the high directivity.

### Physical origin of the high directivity

**Figure 5:**
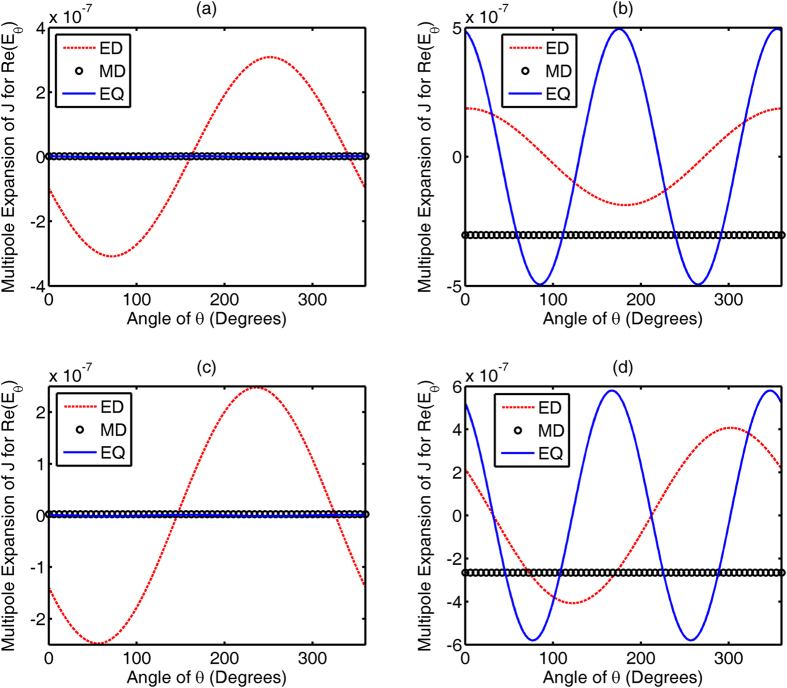
Decomposed real parts of the far-field *E*_θ_ component radiated by multipolar moments of equivalent electric current, including electric dipolar (ED), magnetic dipolar (MD), and electric quadrupolar (EQ) moments. (**a,c**) show the multipolar contributions from the metallic feed; (**b,d**) show the multipolar contributions from the dielectric director and reflector, respectively. (**a,b**) correspond to the feed-director system; (**c,d**) correspond to the feed-reflector system.

**Figure 6:**
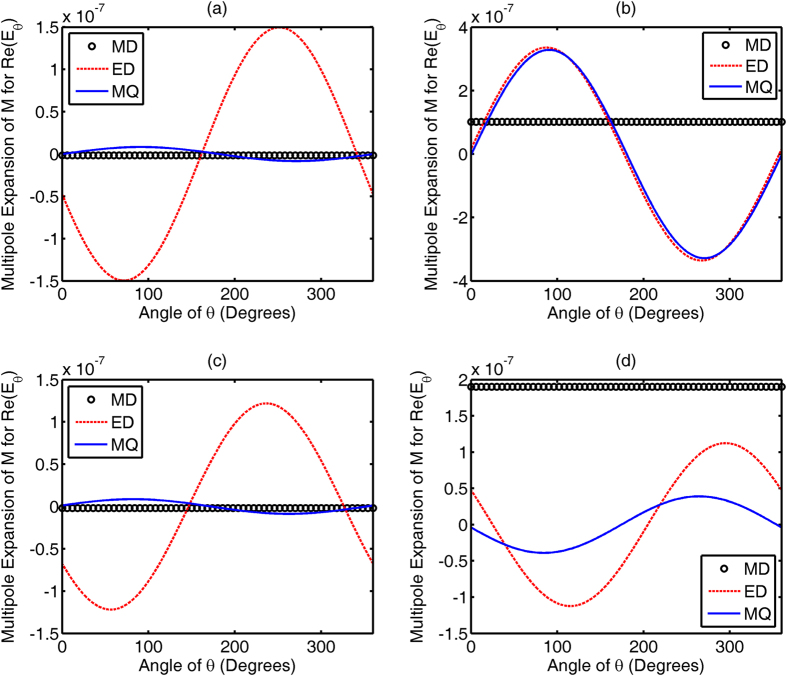
Decomposed real parts of the far-field *E*_θ_ component radiated by multipolar moments of equivalent magnetic current, including magnetic dipolar (MD), electric dipolar (ED), and magnetic quadrupolar (MQ) moments. (**a,c**) show the multipolar contributions from the metallic feed; (**b,d**) show the multipolar contributions from the dielectric director and reflector, respectively. (**a,b**) correspond to the feed-director system; (**c,d**) correspond to the feed-reflector system.

Equivalent electric and magnetic currents on the metallic feed and dielectric elements are responsible for the far-field radiation pattern of the nonlinear nanoantenna. Using the multipole expansion technique^12^ to analyze the simplified two-sphere system (See Supplementary Note 1), we decompose the electric field at the far-field zone in terms of multipolar moments of equivalent currents. At the *yoz* plane concerned, the *E*_θ_ component is three orders of magnitude larger than the *E*_φ_ component. Therefore, the real part of the *E*_θ_ component, i.e. Re(*E*_θ_), is decomposed in terms of multipolar contributions to examine the phase information from the two antenna elements. Figures 5 and 6 show the multipolar contributions with respect to the equivalent electric current and equivalent magnetic current, respectively. It should be noted that Figs 5 and 6 depict the decomposed Re(*E*_θ_) components radiated by corresponding multipoles, including the electric dipole, magnetic dipole, electric quadrupole and magnetic quadrupole. After comparatively studying Figs 5(a,c) and 6(a,c), revealing a strong electric dipolar field, together with a weak magnetic quadrupolar field, leads to the slightly distorted dipolar pattern for second harmonic radiation from the metallic feed [See Fig. 1(b)]. Moreover, incorporation of the reflector or director elements only affects the amplitude (not the phase) of equivalent currents on the surface of the feed. However, after examining Figs 5(b,d) and 6(b,d), there are a significant phase shift and even a phase flip in the electric dipolar and magnetic quadrupolar moments of the dielectric element, which are induced by the metallic feed. When the dielectric element functions as a reflector, the multipolar (electric dipolar and magnetic quadrupolar) moments of metal feed and the induced counterparts of dielectric element constructively interfere in the backward direction. When the dielectric element functions as a director, the constructive interference occurs in the forward direction. The mutual couplings between the second harmonic currents (from the metal feed) and induced currents (from the reflector and director) cause a constructive interference of second harmonic waves in one direction and a destructive interference in the opposite.

### Generalization to other geometries

**Figure 7:**
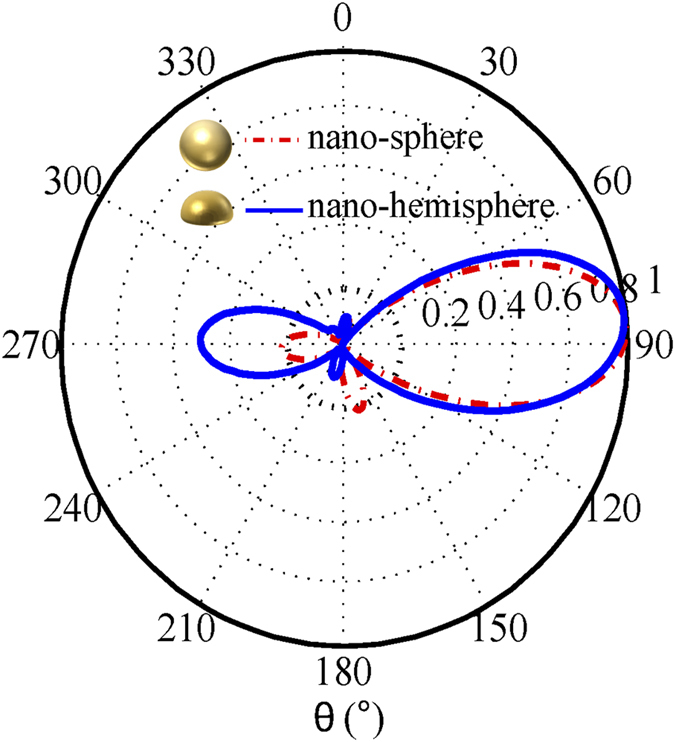
Radiation patterns of the nonlinear spherical and hemispherical nanoantennas at the *yoz* plane. The geometric configuration of the spherical nanoantenna has been given in the caption of Fig. 2. Regarding the hemispherical antenna, the optimized radii for the feed, reflector, and director are 20 nm, 100 nm and 80 nm, respectively. The spacing between the metallic feed and reflector are 80 nm. The spacing between the metallic feed and director are 90 nm.

Other non-spherical nanoparticles could be adopted as building blocks of the nonlinear Yagi-Uda nanoantenna as well. According to the selection rule, second harmonic radiation from an object is strictly zero along the incident ± *z* direction, if the projection of the object onto the *xoy* plane is centrosymmetric. Beyond the spherical antenna discussed above, a hemispherical antenna was also designed to demonstrate the concept. The elements are realized with a flat bottom side and are therefore easier to be deposited on a substrate. The hemispherical antenna also gains a high directivity for second harmonic generation as presented in Fig. 7.

In conclusion, we have proposed a novel nonlinear Yagi-Uda nanoantenna concept to manipulate the second harmonic radiation from a metallic nanosphere feed element. Exploiting the selection rule of second harmonic radiation and the mutual coupling between antenna elements, a unidirectional second harmonic radiation with a high directivity can be spectrally and spatially separated from the incident fundamental pump. The work is of great importance to nonlinear signal detection and sensing.

## Methods

According to Love’s equivalence principles^42^, fundamental fields can be obtained by the surface equivalent currents via dyadic Green’s functions.


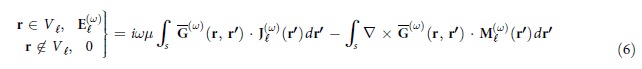



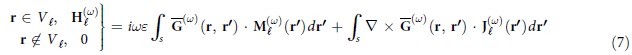


where 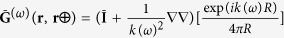 is the dyadic Green’s function at the fundamental frequency and 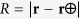. Here, 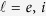 denote the exterior and interior regions of the object, respectively; and 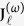 and 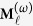 are the equivalent electric and magnetic currents placed on the outer (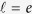) and inner (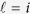) sides of the surface of a nanostructure. The boundary conditions at the surface are given by


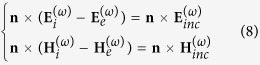


where 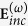 and 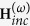 are the incident electric and magnetic fields, respectively. The incident fields include the fundamental pump fields and the scattered fields from other nanoscatterers, which are incident on the considered nanostructure. A boundary element method^16^,^39^,^40^ is employed to solve fundamental fields with equations (6, 7, 8).

After computing the fundamental fields, the second harmonic sources can be expressed by the second harmonic polarization.


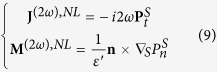


where **n** is the outer normal vector of the surface. 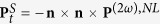, 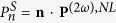, and 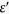 is the selvedge region permittivity^39^. The second harmonic polarization can be obtained by the fundamental fields and second-order nonlinear susceptibility, i.e. 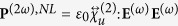.

Considering that the surface contribution will dominate the second harmonic radiation from a metallic nanostructure, using the Love’s equivalence principles again, the second harmonic field can also be connected to the surface equivalent currents via dyadic Green’s functions.


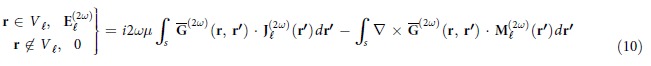



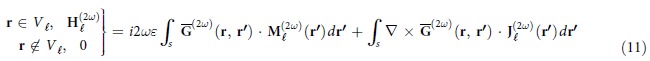


where 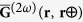 is the dyadic Green’s function at the second harmonic frequency. 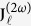 and 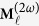 are the equivalent electric and magnetic currents at the second harmonic frequency. In view of the existing second harmonic sources at the surface, the corresponding boundary conditions are given by


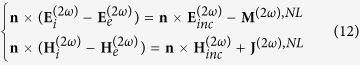


where 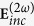 and 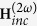 are the scattered electric and magnetic fields from other nanoscatterers, which are incident on the metallic nanostructure at the second harmonic frequency. The second harmonic sources 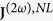 and 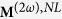 have been computed with equation (9). By using the equivalence principles as shown in equations (10, 11) and the boundary conditions as shown in equation (12), the equivalent currents 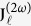 and 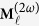 and thus the second harmonic fields can be solved.

Next, it is necessary to solve the fundamental fields once again to capture the pump depletion effect. The boundary conditions in equation (8) will be modified as


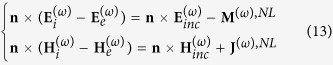


where the first-order nonlinear sources 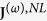 and 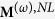 are incorporated to take into account the back coupling from the second harmonic fields to the fundamental fields. Using the updated fundamental fields, the second harmonic fields can be updated as well. This self-consistent process is then repeated iteratively until both fundamental and second harmonic fields converge.

From the boundary element method described, multiple scattering between feed, reflector, and director of the proposed nonlinear antenna at both the fundamental and second harmonic frequencies are modeled. It shall be noted that there is no nonlinear source at the surface of dielectric reflector and director. Considering high density gradient and strong delocalization of electrons, together with a plasmonic enhancement at the metal surface^43^,^44^, the second-order nonlinearity of the TiO_2_ nanoparticle is ignorable compared to the gold nanoparticle. Figure S1 shows the power conversion efficiency of the nonlinear nanoantenna. Under high input power conditions, the pump depletion effect should be taken into account.

The bulk linear susceptibility 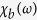 of gold is taken from reference^45^. Surface nonlinear susceptibilities are set as: 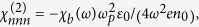 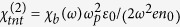 , and 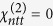, where *e* is the elementary charge, *n*_0_ is the number density of conduction electrons, and 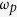 is the plasma frequency. In fact, the shape of the radiation pattern of a metallic nanosphere only relies on 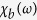 and the ratio of 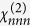 to 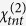. The complex refractive indices of TiO_2_^46^ are 2.446 at 780 nm and 2.872 + 0.007i at 390 nm. Centrosymmetric TiO_2_ material is almost an insulator at 390 nm because of a weak absorption. Consequently, the second-order nonlinearity of TiO_2_ can be ignored in comparison with gold.

## Additional Information

**How to cite this article**: Xiong, X.Y.Z. *et al.* Compact Nonlinear Yagi-Uda Nanoantennas. *Sci. Rep.* **6**, 18872; doi: 10.1038/srep18872 (2016).

## Supplementary Material

Supplementary Information
