# Supplementary material for: Compact Nonlinear Yagi-Uda Nanoantennas
Source: Sci Rep. 2016 Jan 7;6:18872. doi: 10.1038/srep18872 (PMC4703990; doi:10.1038/srep18872)
Supplement: Supplementary Information [file srep18872-s1.pdf]

Compact Nonlinear Yagi-Uda Nanoantennas

Xiaoyan Y.Z. Xiong [1], Li Jun Jiang [1], Wei E.I. Sha [1], Yat Hei Lo [1], and Weng Cho Chew [2]

[1] Department of Electrical and Electronic Engineering, The University of Hong Kong, Pokfulam Road, Hong Kong

[2] Department of Electrical and Computer Engineering, University of Illinois, Urbana-Champaign, Illinois 61801, USA

Emails: ljiang@eee.hku.hk (L.J. Jiang) and wsha@eee.hku.hk (W.E.I. Sha)

Supporting Information

Supplementary Note 1: Multipole Expansion for Equivalent Currents

According to Huygens' principle, electric field can be represented by the Green's tensor $\overline{\mathbf{G}}$

$$\mathbf{E} = i\omega\mu \int \overline{\mathbf{G}}\left(\mathbf{r},\mathbf{r}'\right) \cdot \mathbf{J}\left(\mathbf{r}'\right) d\mathbf{r}' - \int \nabla \times \overline{\mathbf{G}}\left(\mathbf{r},\mathbf{r}'\right) \cdot \mathbf{M}\left(\mathbf{r}'\right) d\mathbf{r}' \tag{1}$$

where $\overline{\mathbf{G}}\left(\mathbf{r},\mathbf{r}'\right) = (\overline{\mathbf{I}} + \frac{\nabla\nabla}{k^2}) g\left(\mathbf{r},\mathbf{r}'\right)$ and $g\left(\mathbf{r},\mathbf{r}'\right) = \frac{\exp(ik|\mathbf{r}-\mathbf{r}'|)}{4\pi|\mathbf{r}-\mathbf{r}'|}$.

In the far-field region ($k\mathbf{r} \gg 1$), it is sufficient to approximate

$$|\mathbf{r} - \mathbf{r}'| \approx r - \hat{\mathbf{r}} \cdot \mathbf{r}' \tag{2}$$

Then electric field is

$$\begin{aligned}\mathbf{E} &= i\omega\mu \frac{e^{ikr}}{4\pi r}\left(\hat{\theta}\hat{\theta} + \hat{\phi}\hat{\phi}\right) \cdot \int \mathbf{J}\left(\mathbf{r}'\right) e^{-ik\hat{\mathbf{r}}\cdot\mathbf{r}'} d\mathbf{r}' - ik\frac{e^{ikr}}{4\pi r}\hat{\mathbf{r}} \times \int \mathbf{M}\left(\mathbf{r}'\right) e^{-ik\hat{\mathbf{r}}\cdot\mathbf{r}'} d\mathbf{r}' \\ &= ik\eta \frac{e^{ikr}}{4\pi r}\left[\left(\hat{\theta}\hat{\theta} + \hat{\phi}\hat{\phi}\right) \cdot \int \mathbf{J}\left(\mathbf{r}'\right) e^{-ik\hat{\mathbf{r}}\cdot\mathbf{r}'} d\mathbf{r}' - \hat{\mathbf{r}} \times \int \widetilde{\mathbf{M}}\left(\mathbf{r}'\right) e^{-ik\hat{\mathbf{r}}\cdot\mathbf{r}'} d\mathbf{r}'\right], \ k\mathbf{r} \gg 1\end{aligned} \tag{3}$$

where $\widetilde{\mathbf{M}} = \mathbf{M}/\eta$ and η is the wave impedance of free space.

If the source dimensions are small compared to a wavelength, we could use Taylor series to expand the phasor term $e^{-ik\hat{\mathbf{r}}\cdot\mathbf{r}'}$ in (3)

$$e^{-ik\hat{\mathbf{r}}\cdot\mathbf{r}'} = \sum_n \frac{(-ik)^n}{n!}(\hat{\mathbf{r}} \cdot \mathbf{r}')^n \approx 1 + (-ik)(\hat{\mathbf{r}} \cdot \mathbf{r}') \tag{4}$$

where the first-order Taylor expansion is employed. Consequently, the current integral is approximated as

$$\int \mathbf{J}\left(\mathbf{r}'\right) e^{-ik\hat{\mathbf{r}}\cdot\mathbf{r}'} d\mathbf{r}' \approx \int \mathbf{J}\left(\mathbf{r}'\right) d\mathbf{r}' + (-ik)\int \mathbf{J}\left(\mathbf{r}'\right)(\hat{\mathbf{r}} \cdot \mathbf{r}') d\mathbf{r}' \tag{5}$$

The first term of (5) is related to the electric dipole radiation

$$\int \mathbf{J}\left(\mathbf{r}'\right) d\mathbf{r}' = -\int \mathbf{r}'\left(\nabla' \cdot \mathbf{J}\left(\mathbf{r}'\right)\right) d\mathbf{r}' = -i\omega \int \mathbf{r}'\rho(\mathbf{r}') d\mathbf{r}' = -i\omega \mathbf{p}^e \tag{6}$$

where $\mathbf{p}^e = \int \mathbf{r}'\rho(\mathbf{r}')d\mathbf{r}'$ is the electric dipole moment. The second term of (5) correlates to the magnetic dipole and electric quadrupole radiations.

First, we can split the integrand into a symmetric and antisymmetric parts

$$(\hat{\mathbf{r}} \cdot \mathbf{r}')\mathbf{J} = \frac{1}{2}\left[(\hat{\mathbf{r}} \cdot \mathbf{r}')\mathbf{J} + (\hat{\mathbf{r}} \cdot \mathbf{J})\mathbf{r}'\right] + \frac{1}{2}(\mathbf{r}' \times \mathbf{J}) \times \hat{\mathbf{r}} \tag{7}$$

Then, we find the antisymmetric part can be expressed by magnetic dipole moment

$$\int \frac{1}{2}(\mathbf{r}' \times \mathbf{J}) \times \hat{\mathbf{r}} d\mathbf{r}' = -\hat{\mathbf{r}} \times \mathbf{m} \tag{8}$$

where $\mathbf{m} = \frac{1}{2}\int \mathbf{r}' \times \mathbf{J}(\mathbf{r}')\, d\mathbf{r}'$ is the magnetic dipole moment. Next, the symmetric part can be expressed by electric quadrupole moment tensor

$$\begin{aligned} &\frac{1}{2}\int \left[(\hat{\mathbf{r}} \cdot \mathbf{r}')\mathbf{J}(\mathbf{r}') + (\hat{\mathbf{r}} \cdot \mathbf{J}(\mathbf{r}'))\mathbf{r}'\right] d\mathbf{r}' \\ &= \frac{1}{2}\int (\mathbf{r}'\mathbf{J}(\mathbf{r}') + \mathbf{J}(\mathbf{r}')\mathbf{r}') \cdot \hat{\mathbf{r}} d\mathbf{r}' \\ &= \frac{-i\omega}{2}\int \rho(\mathbf{r}')\mathbf{r}'\mathbf{r}' d\mathbf{r}' \cdot \hat{\mathbf{r}} = \frac{-i\omega}{6}\overline{\mathbf{Q}}^e \cdot \hat{\mathbf{r}} \end{aligned} \tag{9}$$

where $\overline{\mathbf{Q}}^e = \int (3\mathbf{r}'\mathbf{r}' - r'^2\overline{\mathbf{I}})\rho(\mathbf{r}')d\mathbf{r}'$ is the electric quadrupole moment tensor. Finally, the current integral can be rewritten as a summation of electric dipole, magnetic dipole, and electric quadrupole terms

$$\begin{aligned} \int \mathbf{J}(\mathbf{r}')\, e^{-ik\hat{\mathbf{r}}\cdot\mathbf{r}'} d\mathbf{r}' &\approx \mathbf{J}_{ed} + \mathbf{J}_{md} + \mathbf{J}_{eq} \\ &= -i\omega\mathbf{p}^e + ik\hat{\mathbf{r}} \times \mathbf{m} - \frac{k\omega}{6}\overline{\mathbf{Q}}^e \cdot \hat{\mathbf{r}} \end{aligned} \tag{10}$$

Based on the dual principle, we have a similar expression for the magnetic current integral, i.e.

$$\begin{aligned} \int \mathbf{M}(\mathbf{r}')\, e^{-ik\hat{\mathbf{r}}\cdot\mathbf{r}'} d\mathbf{r}' &\approx \mathbf{M}_{md} + \mathbf{M}_{ed} + \mathbf{M}_{mq} \\ &= -i\omega\mathbf{p}^m + ik\hat{\mathbf{r}} \times \mathbf{m}^e - \frac{k\omega}{6}\overline{\mathbf{Q}}^m \cdot \hat{\mathbf{r}} \end{aligned} \tag{11}$$

Supplementary Note 2: Power Conversion Efficiency of the Nonlinear Nanoantenna

The power conversion efficiency (η) of the nonlinear nanoantenna is defined as the output radiation power (P_o) at the second-harmonic frequency over the input power at the fundamental frequency (P_i).

$$\eta = \frac{P_o}{P_i} = \frac{\int_s \mathrm{Re}\left[\mathbf{E}^s(2\omega) \times \mathbf{H}^s(2\omega)\right] \cdot d\mathbf{S}}{\int_s \frac{|\mathbf{E}^i(\omega)|^2}{Z} dS} \tag{12}$$

where $Z = 120\pi$ is the wave impedance of free space. The effective focus area of laser is set to be 2 um $\times$ 2 um. **Figure S1** shows the power conversion efficiency of the nonlinear nanoantenna. From the figure, under a high input power ($P_i > 10^5$ W) condition, pump depletion effect should be taken into account.

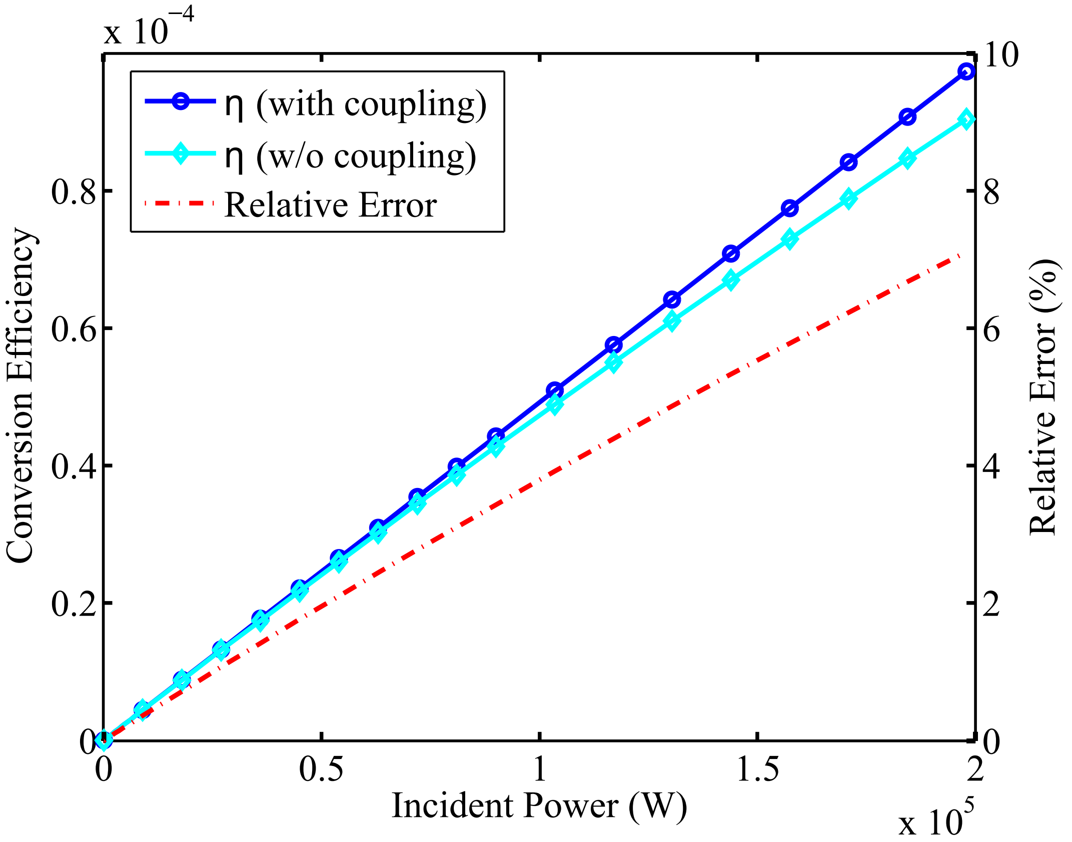

Figure 1: Power conversion efficiency of the nonlinear spherical nanoantenna. The undepleted pump approximation (w/o coupling case) is inaccurate under a high input power condition.
